# Clinical Value of Mean Platelet Volume to Platelet Ratio (MPR) in Distinguishing Mass-Forming Chronic Pancreatitis and Pancreatic Cancer

**DOI:** 10.3390/diagnostics13193126

**Published:** 2023-10-04

**Authors:** Han-Xuan Wang, Yu-Lin Li, Jin-Can Huang, You-Wei Ma, Ren Lang, Shao-Cheng Lyu

**Affiliations:** Department of Hepatobiliary and Pancreaticosplenic Surgery, Beijing Chaoyang Hospital, Capital Medical University, Beijing 100020, China

**Keywords:** pancreatic cancer, mass-forming chronic pancreatitis, mean platelet volume, platelet count, differential diagnosis

## Abstract

Background: Correctly distinguishing mass-forming chronic pancreatitis (MFCP) from pancreatic cancer (PC) is of clinical significance to determine optimal therapy and improve the prognosis of patients. According to research, inflammation status in PC is different from that in MFCP. Mean platelet volume/platelet ratio (MPR) is a platelet-related inflammation index which has been proven to be valuable in the diagnosis and prognosis of various malignant cancers due to the change in mean platelet volume and platelet count under abnormal inflammatory conditions caused by tumors. Thus, we conducted this study to investigate the clinical value of MPR in distinguishing MFCP from PC. Methods: We retrospectively analyzed the data of 422 patients who were suspected to have PC during imaging examination at our department from January 2012 to December 2021. Included patients were divided into the PC (*n* = 383) and MFCP groups (*n* = 39), according to their pathological diagnosis. Clinical data including MPR were compared within these two groups and the diagnostic value was explored using logistic regression. The ROC curve between MPR and PC occurrence was drawn and an optimal cut-off value was obtained. Propensity score matching was applied to match MFCP patients with PC patients according to their age and carbohydrate antigen 19-9 (CA19-9). Differences in MPR between groups were compared to verify our findings. Results: The area under the ROC curve between MPR and PC occurrence was 0.728 (95%CI: 0.652–0.805) and the optimal cut-off value was 0.045 with a 69.2% sensitivity and 68.0% accuracy. For all the included patients, MPRs in the MFCP and PC groups were 0.04 (0.04, 0.06) and 0.06 (0.04, 0.07), respectively (*p* = 0.005). In patients with matching propensity scores, MPRs in the MFCP and PC groups were 0.04 (0.03, 0.06) and 0.06 (0.05, 0.08), respectively (*p* = 0.005). Multiple logistic regression in all included patients and matched patients confirmed MPR and CA19-9 as independent risk markers in distinguishing PC. Combining CA19-9 with MPR can increase the sensitivity and accuracy in diagnosing PC to 93.2% and 89.5%, respectively. Conclusion: MPR in PC patients is significantly higher than that in MFCP patients and may be adopted as a potential indicator to distinguish MFCP and PC. Its differential diagnosis capacity can be improved if combined with CA19-9.

## 1. Introduction

Pancreatic cancer (PC) is derived from pancreatic duct epithelial cells and is one of the most common malignant tumors in the digestive system. It has a high degree of malignancy and rapid disease progression, and the 5-year survival rate is only 11% [1]. Mass-forming chronic pancreatitis (MFCP) is a benign disease caused by repeated inflammation of the pancreas resulting in the replacement of normal pancreatic tissue with fibrous tissue. Since the treatment and long-term prognosis of these two diseases are entirely different, correctly identifying these two diseases can enable PC patients to obtain timely surgery and improve their overall prognosis, which has important clinical significance [2]. However, these two different diseases not only have similar imaging manifestations, but also can show similar clinical symptoms and auxiliary examination characteristics in clinical practice, bringing about great difficulties regarding their identification [3]. Currently, the main methods to distinguish MFCP from PC are abdominal enhanced computerized tomography (CT), magnetic resonance imaging (MRI) and other imaging methods [4]. Some have also reported the value of serum testosterone, immunoglobulin G 4 (IgG4) and tumor markers carcino-embryonic antigen (CEA), carbohydrate antigen 19-9 (CA19-9) and carbohydrate antigen 242 (CA242) in differentiating between MFCP and PC [5,6,7]. However, how to distinguish these two diseases effectively remains an important problem to be solved.

It has been reported that systemic inflammation plays an important role in the occurrence and development of PC [8,9]. Systemic inflammatory indicators such as neutrophil-to-lymphocyte ratio (NLR), lymphocyte-to-monocyte ratio (LMR) and systemic immune-inflammation index (SII) have been reported to be related to the prognosis of PC, which may be related to the cytokine-mediated changes in the tumor microenvironment [10,11,12]. Tanţău et al. compared serum marker levels in MFCP and PC patients and found that the C-reactive protein (CRP) level, also a systemic inflammation index, was significantly higher in PC patients, indicating different systemic inflammation levels between MFCP and PC patients [13]. Therefore, indexes that can reflect the systemic inflammation level may be valuable in distinguishing MFCP from PC. Mean platelet volume to platelet count (MPR), a platelet-related inflammation index, has been confirmed to have a close correlation with a variety of malignant tumors. Gong et al. conducted research on PC patients and found that the MPR value in PC patients was significantly higher than that in patients with pancreatic benign tumors, and the long-term prognosis of PC patients with higher MPR was worse, which may be related to the tumor-related hypercoagulable state reflected by a high MPR and massive consumption of platelets caused by advanced tumors [14]. Cho et al. also confirmed that the MPR level in patients with hepatocellular carcinoma was significantly higher than that in healthy people, indicating MPR as a potential diagnostic index for patients with primary hepatocellular carcinoma [15]. Moreover, the MPR level in patients with colorectal cancer, non-small cell lung cancer and esophageal cancer was significantly different from that in healthy people [16,17,18]. Therefore, we hypothesized that MPR may be a potential index to distinguish MFCP from PC patients, but no relevant studies have been reported so far.

This study aims to compare the difference in MPR in MFCP and PC patients, and explore its clinical value in differentiating MFCP and PC.

## 2. Material and Methods

### 2.1. Patients Screening

**Figure 1:**
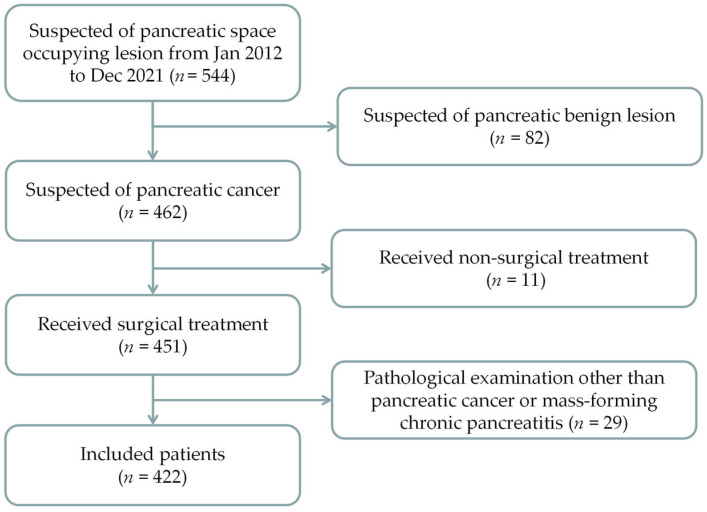
Flow of patients’ inclusion.

The data of patients with pancreatic space-occupying lesions that were admitted to the Hepatobiliary Surgery Department of our hospital from January 2012 to December 2021 were retrospectively analyzed, and patients who met the inclusion and exclusion criteria were included for further analysis (Figure 1).

Inclusion criteria: (1) patients with pancreatic space-occupying lesions admitted to the Hepatobiliary Surgery Department, Beijing Chaoyang Hospital, from January 2012 to December 2021; (2) preoperative imaging examination that indicated PC; (3) those who received surgical treatment and had tumor tissue; (4) postoperative pathological examination that indicated PC or MFCP.

Exclusion criteria: (1) patients who refused to participate in this research; (2) those with incomplete clinical data.

Surgical methods and treatment regimens of the included patients were obtained following the informed consent of patients and their families. The acquisition of clinical data of patients was approved by the Ethics Committee of Beijing Chaoyang Hospital (No. 2020-D.-302, date of approval: 28 May 2020).

### 2.2. Patients Grouping

Included patients were divided into the MFCP group and PC group according to the postoperative pathological diagnosis results, and various indexes were compared between these two groups. Then, the propensity score matching (PSM) method was applied to match MFCP patients with PC patients according to age and preoperative CA19-9 level, and the indexes were compared between these two groups. Subsequently, included patients were further grouped according to the optimal cut-off value of MPR obtained from the receiver operator character curve (ROC) between MPR and PC occurrence and the incidence of PC within these two groups was compared.

### 2.3. Data Collection and Analysis

Preoperative general data (gender, age, initial symptoms, diabetes, previous medical history, etc.), preoperative laboratory examinations (blood routine, blood biochemistry, CEA, CA19-9, etc.), preoperative imaging examinations (abdominal enhanced CT, abdominal enhanced MRI, etc.) and postoperative pathological data of patients were obtained from medical records. The preoperative laboratory examination results were taken from the laboratory examination results within 7 days before surgery. MPR was calculated using the preoperative mean platelet volume (MPV) divided by the platelet count. The differences in preoperative data among different groups of patients were compared.

### 2.4. Statistical Analysis

Measurement data were presented as means ± standard errors of the mean and compared with t tests if conforming to a normal distribution. Measurement data with non-normal distribution were presented as median (interquartile range) and were compared using the rank sum test. Fisher analysis was applied in the comparison of categorical data if the theoretical frequency was less than 1 or the sample size was less than 40; otherwise, Chi-square analysis was selected for the comparison. Overall risk was calculated using the logistic regression model. All statistical analyses were performed using SPSS (version 26.0; IBM Corporation, Armonk, NY, USA), with two-sided *p* values < 0.05 considered to be significant.

## 3. Results

### 3.1. General Data

A total of 422 patients were included in our study, including 259 males and 163 females (male–female = 1.6:1, age 62.4 ± 10.9 years). The initial symptoms included 189 cases of abdominal pain, 146 cases of jaundice, 30 cases of atypical gastrointestinal symptoms, and the remaining 57 cases were diagnosed during physical examination. Among all patients, 126 (29.9%) had a history of diabetes and 4 (0.9%) had a family history of PC. Among the 146 patients with jaundice, 87 patients received preoperative jaundice reduction treatment, including 19 patients with endoscopic retrograde cholangiopancreatography (ERCP) and 68 patients with percutaneous transhepatic choledochus drainage (PTBD).

### 3.2. Postoperative Pathological Examination Results

All included patients received surgical treatment and tumor tissues were obtained intraoperatively. Postoperative pathological examination confirmed that 383 patients had PC, including 236 cases (61.6%) at the pancreatic head, 51 cases (13.3%) at the pancreatic neck, 83 cases (21.7%) at the pancreatic body and tail, and the remaining 13 cases (3.4%) at the uncinate process of the pancreas. A total of 39 patients had MFCP, including 29 cases (74.4%) at the pancreatic head, 1 case (2.6%) at the pancreatic neck, and 9 cases (23.0%) at the pancreatic body and tail.

### 3.3. Grouping Condition

Included patients were divided into an MFCP group (*n* = 39) and a PC group (*n* = 383) according to their postoperative pathological diagnosis. In MFCP and PC groups, 20 and 169 (51.3% vs. 44.1%, *p* = 0.392) patients showed abdominal pain, 9 and 139 (23.1% vs. 36.3%, *p* = 0.099) showed jaundice and 5 and 25 (12.8% vs. 6.5%, *p* = 0.145) patients showed atypical gastrointestinal symptoms as their initial symptoms, respectively, indicating no statistical difference in the initial symptoms between the MFCP and PC groups. The comparison of preoperative general data within these two groups is shown in Table 1. We can observe that there were statistically significant differences in gender, age, preoperative MPV, preoperative total bilirubin (TB), preoperative CA19-9 and MPR between the MFCP and PC groups (*p* < 0.05). The age, MPV, TB, CA19-9 and MPR in the PC group were significantly higher than those in the MFCP group. The above indicators (gender, age, MPV, TB, CA19-9 and MPR) were further included in the logistic regression model as independent variables, and PC occurrence was taken as the dependent variable. As shown in Table 2, age (*OR* = 1.045, 95%CI: 1.009–1.082), CA19-9 (*OR* = 1.026, 95%CI: 1.010–1.042) and MPR (*RR* = 1.210, 95%CI: 1.002–1.461) were independent risk markers for PC occurrence. The older the patient, the higher the CA19-9 and MPR, and the higher the risk of PC occurrence.

### 3.4. Condition of Matching MFCP Patients with PC Patients

In order to further verify the value of MPR in predicting PC occurrence, we applied the PSM method to match the included MFCP patients with the included PC patients according to their age and preoperative CA19-9 level. A total of 35 MFCP patients were successfully paired with PC patients. As shown in Table 3, we can see that MPR in the MFCP group was still significantly lower than that in the PC group despite there being no statistical difference in age and CA19-9 level between the matched groups (*p* = 0.001), confirming MPR as a risk marker of PC occurrence independent of age and CA19-9.

### 3.5. Value of MPR in Distinguishing MFCP from PC

**Figure 2:**
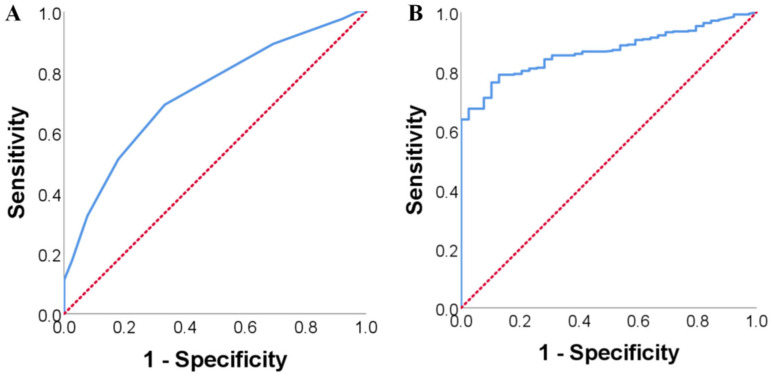
ROC curve between MPR, CA19-9 and PC occurrence. (**A**): ROC curve between MPR and PC occurrence; (**B**): ROC curve between CA19-9 and PC occurrence (MPR: mean platelet volume to platelet count; CA19-9: carbohydrate antigen 19-9; PC: pancreatic cancer; ROC: receiver operator characteristic curve).

The ROC curve between preoperative MPR and PC occurrence was drawn and is shown in Figure 2A. The area under the curve (AUC) was 0.728 (95%CI: 0.652–0.805) and the optimal cut-off value was 0.045. Its sensitivity, specificity and accuracy in predicting PC occurrence were 69.2%, 66.7% and 68.0%, respectively. According to the optimal cut-off value, the included patients were further divided into a high MPR group (MPR ≥ 0.045, *n* = 282) and low MPR group (MPR < 0.045, *n* = 140), and the incidence of PC in both groups was calculated. There were 17 cases of MFCP and 265 cases of PC in the high MPR group and 22 cases of MFCP and 118 cases of PC in low MPR group (*p* = 0.001), indicating that MPR is a potential marker for distinguishing MFCP from PC. Meanwhile, the ROC curve between preoperative CA19-9 and PC occurrence was also drawn and is shown in Figure 2B, according to which the optimal cut-off value of CA19-9 was calculated. The AUC was 0.868 (95%CI: 0.829–0.906) and the optimal cut-off value was 32.55 U/mL. Its sensitivity, specificity and accuracy in predicting PC occurrence were 78.9%, 87.2% and 79.6%, respectively.

### 3.6. Value of Combining MPR with C19-9 in Distinguishing MFCP from PC

Since both high CA19-9 and MPR levels were independent risk markers for PC occurrence, we introduced a combined index of MPR and CA19-9 to further explore their value in differentiating MFCP from PC. According to the previously calculated cut-off values of CA19-9 and MPR, CA19-9 ≥ 32.55 U/mL was defined as positive CA19-9 and MPR ≥ 0.045 was defined as positive MPR. The combined index was defined as positive if there was at least one positive index in CA19-9 and MPR, while the combined index was defined as negative if both CA19-9 and MPR were negative. Among the included patients, a positive combined index was observed in 357 PC patients and 18 MFCP patients, and negative combined index was observed in 26 PC patients and 21 MFCP patients. The sensitivity, specificity and accuracy of the positive combined index in predicting PC were 93.2%, 53.8% and 89.5%, respectively. Since the sensitivity and accuracy of MPR combined with CA19-9 in distinguishing PC from MRCP were superior to applying CA19-9 and MPR separately, this result suggested that MPR combined with CA19-9 could serve as an effective index in distinguishing MFCP from PC.

## 4. Discussion

Since Virchow first described the possible relationship between inflammation and tumorigenesis, increasing studies have confirmed the close correlation between inflammation and tumors, which is related to the occurrence and progression of various tumors including PC [19,20,21]. Among the known risk factors for PC, obesity and diabetes may cause PC through chronic systemic metabolic inflammation, while chronic pancreatitis may induce PC through local chronic inflammation [22,23]. Moreover, the inflammatory status is abnormal in PC patients. The local infiltration of inflammatory cells in the pancreas mediates the formation of an immunosuppressive microenvironment and the abnormal expression of pancreatic cancer-related cytokines that may lead to systemic inflammatory status changes [8,21,24]. Although MFCP patients may also suffer from abnormal systemic inflammation, current studies have shown that CRP levels and inflammation-related cytokines, interleukin-6 (IL-6), interleukin-10 (IL-10) and interleukin-17 (IL-17), in PC patients were significantly higher than those in MFCP patients, indicating different extents of systemic inflammation between PC patients and MFCP patients. These findings suggest the potential value of inflammatory markers in differentiating MFCP from PC [13].

As a platelet-related inflammation index, MPR has been reported to have certain value in predicting the occurrence and prognosis of a variety of tumors. After retrospectively analyzing PC patients, benign pancreatic tumor patients and healthy controls, Gong et al. found a significantly higher MPR level in PC patients compared with that in benign pancreatic tumor patients and healthy controls, which was consistent with the findings of our study and confirmed the correlation between high MPR and PC occurrence [14]. However, the effect of MPR level on tumorigenesis and progression is heterogeneous among different tumors. In colorectal cancer, non-small-cell lung cancer, esophageal cancer and renal cell carcinoma, a low MPR level may indicate tumorigenesis, metastasis and poor prognosis [16,17,18,25,26]. An abnormal local inflammatory status and platelet production caused by the tumor may be the potential reasons for this, according to researchers. In terms of the different variation tendency of MPR among tumors, no relevant studies have been published so far to explain its mechanism. However, considering the different risk of tumor-related thrombosis in different cancer types, the different degree of thrombocytopenia caused by tumor-induced thrombosis may be one of the potential causes of MPR heterogeneity [27].

MPR is the ratio of MPV to platelet count; therefore, both an increase in MPV and a decrease in platelet count can significantly affect the MPR value. MPV is an important platelet morphological index which is closely related to the inflammatory response of patients. In recent years, MPV has been found to have a certain clinical value in differentiating between benign and malignant tumors. Karaman et al. compared the MPV levels in non-functioning pancreatic neuroendocrine tumor patients and PC patients and found that the MPV levels of PC patients were significantly higher than those of non-functioning pancreatic neuroendocrine tumor patients [28]. In the current research, the comparison between the PC and MFCP groups further confirmed the finding that PC patients had a higher MPV than those with benign pancreatic diseases. We considered that this result may be related to abnormal IL-6 release in PC patients. Previous studies have reported that IL-6 could significantly increase the MPV of platelets [29]. It has also been confirmed that the level of IL-6 in PC patients was significantly elevated compared to that in patients with chronic pancreatitis [13]. This may explain the reason why MPV in PC patients is higher than that in MFCP patients. Moreover, PC is not only prone to cause the occurrence of tumor-related thrombosis, but it is also able to activate platelets and enhance the invasion and metastasis capacity of PC cells, resulting in an abnormal depletion of platelets and a decreased platelet count due to tumor factors in PC patients [30]. An increased MPV and decreased platelet count in PC patients may be potential reasons for the abnormal increase in MPR in PC patients, and may be one of the potential mechanisms of its ability to distinguish PC and MFCP.

In the current study, we also found that the level of serum CA19-9 in PC patients was significantly higher than that in MFCP patients, confirming that CA19-9 is an independent risk marker for PC occurrence, consistent with previous studies. Yamaguchi et al. compared PC and MFCP patients and found that the CA19-9 level in MFCP patients was lower than 120 U/mL and significantly lower than that in PC patients, indicating that the serum CA19-9 level was of certain value in differentiating MFCP and PC [31]. In terms of the cut-off value of CA19-9 in distinguishing PC and MFCP, we did not apply 37 U/mL as the cut-off value of CA19-9, which is widely adopted in PC screening and diagnosis, since it is the best cut-off value for differentiating PC from other pancreatic lesions and may not function as an optimal cut-off value for distinguishing PC from MFCP. Therefore, we applied the optimal cut-off value calculated using the ROC curve in order to improve the differential diagnosis capacity. However, this proposed optimal cut-off value of CA19-9 in distinguishing PC and MFCP requires further research to fully confirm its differential diagnosis value.

In this study, high CA19-9 and MPR levels were both confirmed as independent risk markers for PC occurrence, so the combination of CA19-9 and MPR may have higher value in differentiating MFCP and PC than applying CA19-9 and MPR separately. According to previous research, MPR combined with tumor markers has already been applied in the differential diagnosis of benign and malignant lesions. Zhang et al. combined MPR with CA125, CA724 and CA19-9, and confirmed that MPR combined with tumor markers could better distinguish colorectal cancer from colorectal polyps than MPR alone, and that this combination was also able to predict the differentiation, nerve invasion and vascular invasion of colorectal cancer [32]. Therefore, we introduced a combined index of MPR and CA19-9. Since both CA19-9 and MPR were independent risk markers for PC occurrence according to our findings, either positive CA19-9 or positive MPR can indicate the possibility of PC occurrence. Therefore, the combined index was defined as positive if there was at least one positive index in MPR and CA19-9. It was confirmed that the combined index had better sensitivity and accuracy than MPR and CA19-9 alone in distinguishing PC from MFCP.

In clinical practice, MFCP and PC have similar symptoms and auxiliary examination characteristics, making them difficult to distinguish. The correct identification of the two diseases is of important clinical significance since it can help surgeons to choose reasonable treatment. MPR can be easily obtained and calculated, making it easy to apply in clinic. At the same time, MPR combined with CA19-9 has a higher sensitivity and accuracy in distinguishing PC from MFCP than CA19-9 and MPR alone; thus, it can better assist surgeons in differentiating these two diseases. Currently, EUS-guided biopsy has been recognized as an important method in PC diagnosis with a high specificity and sensitivity and can be selectively performed before surgery [33]. Our research found that some patients who were clinically diagnosed with PC and received surgeries turned out to have MFCP, indicating the potential benefits of EUS-guided biopsy in these patients. Although the sensitivity and accuracy of MPR in differentiating MFCP and PC were less than for EUS-guided biopsy, MPR can be a potential indicator for clinicians to choose EUS-guided biopsy when patients are clinically diagnosed with PC to avoid unnecessary surgery. Also, according to research, imaging examination methods, including abdominal CT, MRI and PET-CT, remain the most valuable in differentiating MFCP from PC [4,34]. Since all of these imaging examinations have their limitations, multi-modality imaging integrating the results of different examinations can increase the accuracy of differential diagnosis. Ruan et al. demonstrated that multi-modality imaging, including abdominal CT, MRI and PET-CT, can increase the ability of differentiating MFCP from PC, indicating the clinical value of multi-modality imaging in differential diagnosis [35]. MPR combined with CA19-9 can also be integrated into the multi-modality imaging models and can further improve their differential diagnostic ability. Therefore, we consider that our research is of clinical value.

However, this study also has the following shortcomings: First, this study is a retrospective clinical study with a limited sample size. A large-scale prospective study is necessary to further verify the findings. Secondly, this study failed to measure the levels of serum IL-6 and other cytokines in PC and MFCP patients, so it is impossible to further explore the underlying mechanism of MPR in differentiating PC from MFCP. Its specific mechanism requires further research.

## 5. Conclusions

MPR may be used as an effective indicator to distinguish PC from MFCP. A high MPR level indicates PC occurrence, which may be related to the abnormal increase in MPV caused by a high level of IL-6 secretion and a decrease in platelet count caused by a large amount of platelet consumption in PC patients. At the same time, CA19-9 is also an effective indicator for the differentiation between MFCP and PC, and the combined application of MPR with CA19-9 can further improve the capacity of distinguishing PC from MFCP.

## Figures and Tables

**Table 1:** Comparison of general data between MFCP group and PC group.

Variables	MFCP Group (*n* = 39)	PC Group (*n* = 383)	*p* Value
Gender (Male/Female)	30 (76.9%)/9 (23.1%)	230 (60.1%)/153 (39.9%)	0.039
Age (y)	56.50 ± 13.90	63.00 ± 10.40	0.000
Diabetes (Yes/No)	9 (23.1%)/30 (76.9%)	117 (30.5%)/266 (69.5%)	0.331
Preoperative jaundice reduction treatment (Yes/No)	6 (15.4%)/33 (84.6%)	81 (21.1%)/302 (78.9%)	0.397
BMI (kg/m^2^)	22.37 ± 3.15	23.01 ± 3.31	0.246
WBC (×10^9^/L)	6.16 (5.03, 7.22)	5.80 (4.70, 7.10)	0.627
PLT (×10^9^/L)	233.00 (170.00, 329.00)	204.00 (165.00, 259.00)	0.060
MPV (fl)	10.50 (9.80, 11.40)	10.90 (10.20, 11.70)	0.034
ALB (g/L)	38.50 ± 6.00	37.60 ± 5.30	0.302
ALT (U/L)	25.00 (18.00, 42.00)	32.0 (16.0,0 85.00)	0.216
TB (μmol/L)	13.60 (8.40, 22.90)	18.10 (10.20, 119.90)	0.016
CEA (ng/mL)	1.59 (1.22, 3.48)	2.50 (1.30, 4.40)	0.092
CA19-9 (U/mL)	14.20 (4.99, 27.01)	218.90 (43.10, 693.10)	0.000
MPR	0.04 (0.04, 0.06)	0.06 (0.04, 0.07)	0.005

MFCP: mass-forming chronic pancreatitis; PC: pancreatic cancer; BMI: body mass index; WBC: white blood cells; PLT: platelet; MPV: mean platelet volume; ALB: albumin; ALT: alanine aminotransferase; TB: total bilirubin; CEA: carcino-embryonic antigen; CA19-9: carbohydrate antigen 19-9; MPR: mean platelet volume to platelet count.

**Table 2:** Results of multiple logistic regression analysis of PC occurrence.

Variables	*OR*	95% CI	*p* Value
Gender (Male/Female)	0.479	0.200–1.144	0.098
Age (y)	1.045	1.009–1.082	0.014
MPV (fl)	0.974	0.742–1.279	0.852
TB (μmol/L)	1.005	0.998–1.011	0.165
CA19-9 (U/mL)	1.026	1.010–1.042	0.001
MPR	1.210	1.002–1.461	0.047

OR: overall risk; CI: confidential interval; MPV: mean platelet volume; TB: total bilirubin; CA19-9: carbohydrate antigen 19-9; MPR: mean platelet volume to platelet count.

**Table 3:** Comparison of general data between MFCP group and PC group after PSM matching.

Variables	MFCP Group (*n* = 35)	PC Group (*n* = 35)	*p* Value
Gender (Male/Female)	26 (74.3%)/9 (25.7%)	23 (65.7%)/12 (34.3%)	0.434
Age (y)	59.34 ± 11.36	60.31 ± 10.42	0.710
Diabetes (Yes/No)	26 (74.3%)/9 (25.7%)	29 (82.9%)/6 (17.1%)	0.382
Preoperative jaundice reduction treatment (Yes/No)	30 (85.7%)/5 (14.3%)	30 (85.7%)/5 (14.3%)	1.000
BMI (kg/m^2^)	22.46 ± 3.30	22.25 ± 2.91	0.777
WBC (×10^9^/L)	6.28 (5.08, 7.35)	6.10 (4.70, 7.10)	0.694
PLT (×10^9^/L)	233.00 (170.00, 329.00)	214.00 (164.00, 275.00)	0.229
MPV (fl)	10.59 ± 1.11	10.96 ± 0.97	0.138
ALB (g/L)	37.9 ± 6.03	37.5 ± 4.82	0.745
ALT (U/L)	25.00 (18.00, 38.00)	25.00 (15.00, 119.00)	0.553
TB (μmol/L)	13.60 (8.40, 28.00)	14.20 (9.00, 134.80)	0.414
CEA (ng/mL)	2.35 (1.36, 3.67)	2.60 (1.60, 5.00)	0.194
CA19-9 (U/mL)	14.20 (4.99, 28.88)	16.80 (4.40, 33.30)	0.549
MPR	4.00 (3.00, 5.00)	5.00 (4.00, 8.00)	0.001

MFCP: mass-forming chronic pancreatitis; PC: pancreatic cancer; BMI: body mass index; WBC: white blood cells; PLT: platelet; MPV: mean platelet volume; ALB: albumin; ALT: alanine aminotransferase; TB: total bilirubin; CEA: carcino-embryonic antigen; CA19-9: carbohydrate antigen 19-9; MPR: mean platelet volume to platelet count.

## Data Availability

The datasets used and/or analyzed during the current study are available from the corresponding author on reasonable request.
